# Determinants for participation in a prevention and early detection programme for children and adolescents in Germany: does social background play a role?

**DOI:** 10.1186/s13690-023-01173-5

**Published:** 2023-08-29

**Authors:** Kathrin Krüger, Anne-Marie Lapstich, Katrin Christiane Reber, Stephanie Sehlen, Sebastian Liersch, Christian Krauth

**Affiliations:** 1 Hannover Medical School, Institute for Epidemiology, Social Medicine and Health Systems Research, Carl-Neuberg-Str. 1, 30625 Hanover, Germany; 2 Center for Health Economics Research Hannover (CHERH), Otto-Brenner-Straße 7, 30159 Hanover, Germany; 3 AOK Nordost. Die Gesundheitskasse, Health Services Management, Wilhelmstr. 1, 10963 Berlin, Germany

**Keywords:** Prevention, Early detection, Children, Adolescents, Social background, Germany

## Abstract

Early detection examinations and prevention are particularly important in childhood and adolescence, as certain diseases are already developing and health-related attitudes and behaviour patterns are formed and implemented. Despite the importance of screening and prevention, not all families use the available services and programmes. The aim of this study is to identify factors associated with participation in an early detection and prevention programme for children and adolescents, as well as factors associated with actual uptake of an examination. The analyses are based on questionnaire data of an online survey of participants and non-participants. Descriptive analyses and logistic regression models are conducted on a defined sample (*n* = 1,289). The results show that both groups differ with regard to several factors: age, chronic diseases, federal state, living space, number of siblings, country of birth, migration background, language spoken at home, mother’s occupational status, household income, treatment duration, and trust in treating physician. Regression I shows that participation in the programme is significantly associated with higher age, language spoken at home, mother’s occupational status and greater trust in the treating physician. The latter demonstrates the highest predictive power. Regression II indicates that the actual uptake of an examination among participants is significantly affected by age, federal state and father’s occupational status. Overall, the results of this study show that social background partly plays a role in participation, but that factors such as trust in the treating physician also have a significant impact. For the future, further research on the factors influencing participation in screening and prevention services or programmes for children and adolescents is important in order to develop strategies to overcome existing barriers and thus reach groups that have not been reached yet. In this context, trust in the treating physician and his or her influence on decision-making should in particular be considered.

**Table:** 

**Text box 1. Contributions to the literature**
• This study examines determinants for participation in a prevention and early detection programme for children and adolescents.
• Social background influences participation less than assumed; for example, parental education has no significant impact.
• A high level of trust in the treating paediatrician is a strong predictor of participation.
• Developing strategies to overcome existing barriers and reach underrepresented groups, such as people with a language barrier, is important.
• The influence of trust in the treating physician or paediatrician should be further investigated and included in the development of strategies to increase participation in prevention and early detection.

## Introduction

Early detection examinations and preventive measures are established in the statutory health insurance fonds for various diseases and age groups in Germany and serve to identify and prevent diseases and disorders at an early stage [9]. Despite the importance of screening and prevention, studies show that social background has an influence on participation in screening and prevention programmes. Indeed, a systematic review reports that people with a higher level of education are more likely to undergo cancer screening or preventive health check-up and that people with a higher socio-economic status more often participate in behavioural preventive measures [13]. In the context of screening and prevention, childhood and adolescence are particularly important, as certain diseases already develop and health-related attitudes and behaviour patterns are formed and implemented. Early detection tests can prevent the manifestation of diseases, mitigate consequences and prevent long-term health risks by detecting and treating early stages of diseases [2, 17, 20, 35]. In Germany, the regular early detection programme for children and adolescents includes various age-specific health examinations (known as U-examinations), whose content, timing and structure vary depending on age. U1 to U6 take place between birth and the end of the first 12 months of life and focus on vital bodily functions and general physical and mental development. The U7, U7a, U8 and U9 can then be performed approximately annually until the child enters elementary school, while the J1 follows at the age of 12 to 14. The examinations consist of a physical examination of the child, special preventive examinations for certain age-specific diseases (e.g. U7a for dental development or U9 for speech development) and counselling of the parents [20]. However, due to several studies a social gradient was also found among children and adolescents. They concluded that children and adolescents from families with a low socio-economic status have lower rates of participation in early detection examinations (i.e. U1-U9, J1) offered by the statutory health insurance. It has also been found that adolescents with a migration background participate in the early detection examination J1 (between 12 and 14 years) only half as often as adolescents without a migration background [6, 7, 25, 30, 32].

In Germany, in addition to the regular benefit catalogue covered by all statutory health insurance funds examinations (U1-U9, J1), there are supplementary services offered by individual health insurance funds. The AOK Nordost (AOKNo), a regional health insurance provider in North-eastern Germany in the federal states of Berlin, Brandenburg and Mecklenburg-Western Pomerania (MWP), developed the programme AOK-Junior for the early detection and treatment of diseases in children and adolescents. As part of this health programme, various additional screening examinations and check-ups are offered for different age groups. These include, for example, the preventive examinations U10 (7–8 years), U11 (9–10 years) and J2 (16- 17 years) as well as additional check-ups for skin and lung diseases and interventions for dental health. AOK-Junior is divided into different performance modules that target age-specific diseases. Not all modules are offered in all three federal states (see Table 1). To participate in this health programme, children and adolescents enrol with their paediatrician and receive information and advice on the screening examinations and check-ups from AOKNo and their paediatrician. The participation, satisfaction and effectiveness of the programme was investigated as part of the project "Evaluation of the Paediatric-Centred Integrated Care AOK Junior (EPIVA)", which was funded by the Innovation Fund of the G-BA (01VSF17004) and started in 2018 [21, 16]. This study was prospectively registered on the German Clinical Trials Register (DRKS), a German WHO primary registry, under the registration number: DRKS00015280 on March 18, 2019.

**Table 1:** Performance modules of AOK-Junior (June 2020)

Performance modules	Age
U10	7–8 years
U11	9–10 years
J2	16–17 years
Allergic Rhinitis	0–17 years
Target Agreement on Overweight	0–17 years
Dental Health	9–17 years
Amblyopy Screening^a^	5.-14. and 20.-27. months
Screening for Eating Disorders^a^	7–17 years
Ophthalmologic Early Screening Examination	32.-42. months
Skin Check	2–4 and 13–17 years
Lung Check	6–7 years

^a^offered only in Berlin

The aim of the present analysis is to identify determinants of participation in such a prevention and early detection programme for children and adolescents and to determine whether certain patient groups are not reached. In addition, potential factors influencing the actual utilization of early detection examination or check-up after participating in the AOK-Junior programme will be investigated. For the reasons described above, a special focus is on the influence of social background, i.e. socio-demographic factors, including migration, and socio-economic factors, on participation behaviour. Since the treating paediatrician can enrol children and adolescents in the AOK-Junior programme, variables regarding treatment duration and trust in the treating paediatrician were also considered.

## Methods

### Research questions and study design

The project EPIVA aimed at providing information on the quality of AOK-Junior in order to derive recommendations for further development of the care model. A detailed description of the design of EPIVA has been published elsewhere [21]. The present study investigates (1) who is participating in the programme AOK-Junior, (2) which determinants have an influence on participation in AOK-Junior (socio-demographic determinants including migration, socio-economic determinants and the role of the treating paediatrician), and (3) whether there are factors among those who participate in AOK-Junior that influence actual utilization of the examinations offered. To answer these questions, survey data and administrative claims data, i.e. the accounting data of the AOKNo, are analysed in a cross-sectional design.

### Sample

In 2020, AOKNo insured approximately 250,000 children and young people under 18 years who were entitled to participate in the AOK-Junior programme. 10,800 families with children between 0 and 17 years of age insured by AOKNo were invited to take part in the survey which was conducted in June 2020. A random sample was selected according to participation status in AOK-Junior (yes/no), age (0–5/6–11/12–17 years), gender (male/female) and region (Berlin/Brandenburg/MWP). The letters of invitation were sent by post. They contained an invitation letter from AOKNo and an information letter from Hannover Medical School including a link or respectively QR code to the online questionnaire as well as an individual password. To increase the response rate, reminders were sent out after 2 and 5 weeks. In addition, 30 age-appropriate gifts of a maximum value of € 30 each were raffled off.

While the gross sample was 10,800, the net sample was 10,011, as some invitations could not be delivered due to incorrect addresses. 1,595 families took part in the survey, of which 1,489 gave their consent in analysing and linking survey and claims data. Questionnaires in which no information was provided on the relevant parameters (e.g. educational status, income, country of birth) were excluded from the present analysis, resulting in a final sample size of 1,289.

### Measures

The online questionnaire was offered in four different languages: German, Turkish, Russian and Arabic. Choice of languages was based on the population structure of the three federal states. In total, the questionnaire contained 60 questions; depending on the filter guide, however, fewer questions were displayed. The questionnaire included, inter alia, questions on socio-demographic and socio-economic parameters as well as satisfaction with children’s healthcare and with their paediatrician. With regard to capturing social background, relevant questions of the German Health Interview and Examination Survey (KiGGS) of the Robert Koch Institute (RKI) were used concerning the following items: highest school-leaving qualification of the parents, highest vocational qualification of the parents, occupational status of the parents, household net income, nationality and country of birth of parents and children, languages spoken at home [19]. Some aspects have been summarized here for the analyses. Migration background was categorized according to the country of birth and nationality of the parents and children as follows: none, one-sided (i.e., one parent has a migration background), two-sided (i.e., both parents have a migration background). In terms of school-leaving qualifications of the parent with the highest degree, three categories were formed: no school-leaving qualification and a low-level qualification equivalent to 9 years of schooling were defended as low, an intermediate qualification equivalent to 10 years of schooling corresponded to middle and a qualification in the form of a school graduation certificate or technical diploma equivalent to 12 or 13 years of schooling equalled high. Regarding vocational qualifications of the parent with the highest degree, the following groups were formed: no qualification, completed vocational training and higher degrees from universities and other higher education establishments. No index for socio-economic status was formed. Instead, it was preferred to use the individual indicators of education, occupation and income, as these are more likely to allow conclusions to be drawn about the relevance of, for example, material living conditions, social participation opportunities or health-related attitudes and behaviours [11, 18].

For the present analysis, the questionnaire data were linked with the claims data of the AOKNo, as some characteristics were not collected via the questionnaire. Claims data are the administrative data of the health insurance funds, which include diagnoses and services in the outpatient and inpatient care as well as drug prescriptions and to some extent socio-demographic and socio-economic data. Participation in AOK-Junior and in the individual performance modules (see Table 1) was directly asked in the questionnaire and verified by the claims data.

The variables included in the analyses for the present study are presented in Table 2.

**Table 2:** Included variables

Parameter	Source	Statistical analysis (regression I and II)
**Socio-demographic parameters**		
Age	Claims data	0–5 (0); 6–11 (1); 12–17 (2)
Sex	Claims data	female (0); male (1)
Chronic disease	Claims data	no (0); yes (1)
Federal State	Claims data	Berlin (0); Brandenburg (1); MWP (2)
Region of residence	Claims data	rural (0); semi-urban (1), urban (2)
Living space	Questionnaire	birth parents (0); mother/father and new partner (1); only mother/father (2)
Siblings	Questionnaire	metric
**Migration parameters**		
Country of birth	Questionnaire	Germany (0); others (1)
Migration background	Questionnaire	none (0); one-sided (1); two-sided (2)
Spoken language at home	Questionnaire	only German (0); German and other languages (1); only other languages (2)
**Socio-economic parameters**		
School leaving qualification^a^	Questionnaire	none or low (0); medium (1); high (2)
Vocational qualification^a^	Questionnaire	none (0); completed vocational training (1); higher degrees (2)
Occupational status of mother	Questionnaire	worker (0); employee (1); official (2); self-employed (3); other (4); unemployed (5)
Occupational status of father	Questionnaire	worker (0); employee (1); official (2); self-employed (3); other (4); unemployed (5)
Household net income	Questionnaire	< €1000 (0); €1000-€2000 (1); €2000-€3000 (3); €3000-€4000 (4); ≥ €4000 (5)
**Treating paediatrician**		
Start of treatment with the current paediatrician	Questionnaire	during first year of life (0); later (1)
Trust in the current paediatrician	Questionnaire	very high (0); rather high (1); low/none (2)
**AOK-Junior-parameters**		
Programme participation	Questionnaire and claims data	no (0); yes (1)
Actual uptake of examination/check-up	Questionnaire and claims data	no (0); yes (1)

^a^degree of the parent with the highest degree

### Statistical analysis

Descriptive statistics of the study population were carried out. Categorical variables were reported as absolute numbers and percentages and continuous variables as means. To test the differences between both groups (participants (P) vs. non-participants (NP)), t-test, Chi squared test or Fisher’s exact test were performed as appropriate. Furthermore, binominal logistic regression modelling for the participation in the programme and the uptake of an early detection examination or check-up was performed to identify associations between characteristics of the children or adolescents. The dependent variable for the first regression was the participation in AOK-Junior, and the dependent variable for the second regression was actual uptake of a service. Potential responses were “yes” or “no” in both cases, thus making it dichotomous variables. The following explanatory variables were included in both models respectively: age, sex, federal state, region of residence (urban or rural)*,* living space*,* country of birth*,* migration background*,* spoken language at home*,* school leaving qualification of parent with highest degree*,* vocational qualification of parent with highest degree, occupational status of mother and father*,* household income, start of treatment by current paediatrician, trust in current paediatrician (see Table 2)*.* A *p*-value less than 0.05 was considered statistically significant. Data were analysed using the software IBM Statistical Package for Social Sciences version 27 (SPSS Inc., Chicago, IL/USA).

## Results

### Sample characteristics

Of the 1,289 subjects selected for the present analyses, 711 participate in AOK-Junior, while 578 are non-participants. Various differences can be observed between the two groups regarding socio-demographic parameters, including migration, as well as socio-economic parameters and the treating physician.

#### Socio-demographic parameters

The participants are older (P: 9.0, NP: 8.1 years; *p* = 0.001) and are more likely to have a chronic disease (P: 16.9%, NP: 12.3%; *p* = 0.022). While differences are evident with respect to federal state (*p* = 0.009), there is no significant difference in terms of region, i.e., whether families live in an urban or rural area. However, an influence of the living environment is evident: among the participants, more children and adolescents live with both birth parents (*p* = 0.017) and the number of siblings also differs significantly between both groups (*p* = 0.007) (see Table 3).

**Table 3:** Differences in general characteristics between participants and non-participants

	Participants (*n* = 711)		Non-participants (*n* = 578)		*p*
	*n*	%	*n*	%	
**Age**					**0.005**
0–5 years	248	34.9%	244	42.2%	
6–11 years	232	32.6%	190	32.9%	
12–17 years	231	32.5%	144	24.9%	
*Missing*	*0*		*0*		
**Sex**					0.823
Female	360	50.6%	288	50.0%	
Male	351	49.4%	288	50.0%	
*Missing*	*0*		*2*		
**Chronic disease**					**0.022**
Yes	120	16.9%	71	12.3%	
No	591	83.1%	507	87.7%	
*Missing*	*0*		*0*		
**Federal State**					**0.009**
Berlin	136	19.1%	151	26.2%	
Brandenburg	296	41.6%	226	39.2%	
MWP	279	39.2%	199	34.5%	
*Missing*	*0*		*2*		
**Region of residence**					0.148
Rural	220	30.9%	157	27.2%	
Semi-urban	227	31.9%	177	30.6%	
Urban	264	37.1%	244	42.2%	
*Missing*	*0*		*0*		
**Living space**					**0.017**
Birth parents	518	72.9%	393	68.0%	
Mother or father and new partner	75	10.5%	56	9.7%	
Only mother or father	112	15.8%	114	19.7%	
Other	6	0.8%	15	2.6%	
*Missing*	*0*		*0*		
**Siblings**					**0.007**
no	145	20.7%	130	23.1%	
1	318	45.4%	204	36.2%	
2	136	19.4%	112	19.9%	
3	54	7.7%	63	11.2%	
≥ 4	48	6.8%	54	9.6%	
*Missing*	*10*		*15*		
**Country of birth**					**0.009**
Germany	661	94.0%	517	89.9%	
Other	42	6.0%	58	10.1%	
*Missing*	*8*		*3*		
**Migration background**					**0.002**
None	505	72.7%	359	63.3%	
One-sided	63	9.1%	65	11.5%	
Two-sided	127	18.3%	143	25.2%	
*Missing*	*16*		*11*		
**Spoken language at home**					**0.001**
German	494	69.6%	346	60.0%	
German and other language(s)	176	24.8%	175	30.3%	
Only other language(s)	40	5.6%	56	9.7%	
*Missing*	*1*		*1*		
**School leaving qualification**^**a**^					0.532
None or low	100	14.5%	92	16.8%	
Medium	279	40.4%	214	39.1%	
High	312	45.2%	242	44.2%	
*Missing*	*20*		*30*		
**Vocational qualification**^**a**^					0.261
None	68	10.0%	70	12.9%	
Completed vocational training	362	53.1%	274	50.4%	
Higher degrees^**b**^	252	37.0%	200	36.8%	
*Missing*	*29*		*34*		
**Occupational status of mother**					**< 0.001**
Worker	201	28.9%	162	28.6%	
Employee	293	42.1%	179	31.6%	
Official	16	2.3%	8	1.4%	
Self-employed	19	2.7%	21	3.7%	
Other	71	10.2%	88	15.5%	
Unemployed	96	13.8%	108	19.1%	
*Missing*	*15*		*12*		
**Occupational status of father**					0.380
Worker	285	42.2%	224	41.3%	
Employee	224	33.1%	165	30.4%	
Official	29	4.3%	21	3.9%	
Self-employed	49	7.2%	36	6.6%	
Other	27	4.0%	32	5.9%	
Unemployed	62	9.2%	64	11.8%	
*Missing*	*35*		*36*		
**Household net income**					**0.020**
< €1000	60	9.2%	58	10.7%	
€1000—< €2000	174	26.8%	181	33.3%	
€2000—< €3000	172	26.5%	149	27.4%	
€3000—< €4000	140	21.6%	91	16.7%	
≥ €4000	103	15.9%	65	11.9%	
*Missing*	*62*		*34*		
**Start of treatment with the current paediatrician**					**0.029**
During the first year of life	480	69.8%	364	64.5%	
Later	208	30.2%	200	35.5%	
*Missing*	*23*		*14*		
**Trust in the current paediatrician**					**< 0.001**
Very high	338	48.6%	204	36.6%	
Rather high	305	43.9%	276	49.5%	
Low/none	52	7.5%	78	14.0%	
*Missing*	*16*		*20*		

^a^degree of parent with the highest degree

^b^degrees from universities, higher education establishments and technical colleges

*p* Pearson-Chi-Square

#### Migration parameters

It was detected that participants are more likely to be born in Germany (P: 94.0%, NP: 89.9%; *p* = 0.002) and less likely to have a two-sided migration background (P: 18.3%, NP: 25.2%; *p* = 0.002). There are also differences between the groups concerning the language spoken at home; in the non-participating families, other languages than German are more often spoken at home (P: 5.6%, NP: 9.7%; *p* = 0.001) (see Table 3).

#### Socio-economic parameters

Interestingly, no significant differences could be identified between both groups with regard to school-leaving qualifications or vocational qualifications of the parents. Nevertheless, there is a significant variation in the occupational status of mothers (*p* < 0.001), with more mothers among participants tending to be working as employees (P: 42.1%, NP: 31.6%) and less mothers being unemployed (P: 13.8%, NP: 19.1%), while the proportions of workers, officials and self-employed are similar. In contrast, no significant difference was observed for the father's occupational position. Income, however, differs between the two groups; participating families tend to achieve a higher household income (*p* = 0.020) (see Table 3).

#### Treating paediatrician

The analyses show that the participating children and adolescents are more likely to have been treated by their current paediatrician since their first year of life (P: 69.8%, NP: 64.5%; *p* = 0.029). It also becomes apparent that the participating families more often indicated that their trust in their treating paediatrician is very high (P: 48.6%, NP: 36.6%; *p* < 0.001) (see Table 3).

### Regression I

The binomial logistic regression model is statistically significant, χ^2^(37) = 76.283, *p* < 0.001. Goodness-of-fit was assessed using the Hosmer–Lemeshow-Test, indicating a good model fit, χ^2^(8) = 6.178, *p* > 0.05. Multicollinearity was tested using collinearity statistics. The resulting VIF values for all independent variables are below the threshold of 10 (ranging from 1.01 to 3.38), indicating no observable multicollinearity.

Overall, the regression shows that the variables age (*p* = 0.013) and trust in current paediatrician (*p* = 0.003) have a significant influence on participating in the AOK-Junior programme among our sample (see Table 4). In fact, the higher age groups 6–11 years (OR: 1.408; 95%-CI: 1.023–1.938) and 12–17 years (OR: 1.678; 95%-CI: 1.168–2.411) are associated with participation and with regard to trust in the paediatrician currently treating the child or adolescent, it appears that a very high level of trust compared to a moderate (OR: 0.671; 95%-CI: 0.506–0.891) or low to non-existent (OR: 0.522; 95%-CI: 0.334–0.814) level of trust is a strong predictor of participation. Furthermore, speaking other languages than German at home is associated with a lower probability of participation compared to families that speak German at home (OR: 0.378; 95%-CI: 0.174–0.860). With respect to the occupational status of the mother, being employed as an employee (OR: 1.499; 95%-CI: 1.025–2.194) is significantly associated with participation in AOK-Junior (see Table 4).

**Table 4:** Logistic regression analyses

	Regression I: *Participation in AOK-Junior* (*n* = 1,006; missing: 283; HL-Test χ^2^ (8) = 6,178, *p* > 0.05; Nagelkerke’s *R*^2^ = 0.100)			Regression II: *Uptake of a service* (*n* = 564; missing: 147; HL-Test χ^2^ (8) = 14.555, *p* > 0.05; Nagelkerke’s *R*^2^ = 0.194)		
	*p*	OR	95%-CI	*p*	OR	95%-CI
**Age**						
0–5 years (Ref.)	**0.013**			**0.011**		
6–11 years	**0.036**	1.408	1.023–1.938	0.216	1.441	0.808–2.571
12–17 years	**0.005**	1.678	1.168–2.411	**0.003**	3.270	1.507–7.094
**Sex**						
Female (Ref.) vs. Male	0.679	0.946	0.726–1.232	0.353	1.274	0.764–2.122
**Chronic disease**						
No (Ref.) vs. Yes	0.205	1.294	0.868–1.927	0.555	1.277	0.568–2.870
**Federal State**						
Berlin (Ref.)	0.141			**0.008**		
Brandenburg	0.054	1.629	0.992–2.673	**0.004**	0.175	0.054–0.567
MWP	0.071	1.532	0.964–2.436	**0.002**	0.175	0.058–0.533
**Region of residence**						
Rural (Ref.)	0.880			0.962		
Semi-urban	0.987	0.997	0.711–1.398	0.864	1.053	0.583–1.904
Urban	0.653	1.100	0.726–1.668	0.893	0.949	0.444–2.030
**Living space**						
Birth parents (Ref.)	0.259			0.217		
Mother or father and new partner	0.842	0.953	0.593–1.532	0.313	1.675	0.615–4.560
Only mother or father	0.102	0.711	0.473–1.07	0.109	2.057	0.851–4.971
**Siblings**	0.069	0.912	0.826–1.007	0.659	1.053	0.838–1.323
**Country of birth**						
Germany (Ref.) vs. Others	0.280	0.704	0.373–1.331	0.884	0.904	0.236–3.472
**Migration background**						
None (Ref.)	0.234			0.142		
One-sided	0.403	1.297	0.705–2.386	0.095	0.404	0.140–1.169
Two-sided	0.092	1.733	0.915–3.283	0.947	1.043	0.296–3.674
**Spoken language at home**						
German (Ref.)	0.063			0.181		
German and other language(s)	0.083	0.627	0.369–1.064	0.246	1.790	0.669–4.787
Only other language(s)	**0.020**	0.387	0.174–0.86	0.564	0.626	0.128–3.070
**School leaving qualification**^**a**^						
None or low (Ref.)	0.996			0.182		
Medium	0.943	0.985	0.645–1.503	0.128	1.874	0.835–4.205
High	0.990	0.997	0.635–1.566	0.740	1.156	0.491–2.722
**Vocational qualification**^**a**^						
None (Ref.)	0.952			0.337		
Completed vocational training	0.934	0.979	0.598–1.604	0.159	1.998	0.762–5.237
Higher degrees^**b**^	0.888	1.039	0.612–1.762	0.414	1.542	0.546–4.355
**Occupational status of mother**						
Worker (Ref.)	0.058			0.548		
Employee	**0.037**	1.499	1.025–2.194	0.346	0.715	0.357–1.435
Official	0.052	3.346	0.988–11.332	0.263	0.399	0.080–1.996
Self-employed	0.730	0.868	0.389–1.936	0.695	1.574	0.163–15.179
Other	0.504	0.852	0.533–1.362	0.563	0.758	0.296–1.939
Unemployed	0.658	0.901	0.569–1.428	0.297	1.755	0.610–5.052
**Occupational status of father**						
Worker (Ref.)	0.720			0.213		
Employee	0.198	0.790	0.551–1.131	**0.040**	2.014	1.033–3.924
Official	0.603	0.817	0.382–1.749	0.175	3.080	0.606–15.644
Self-employed	0.967	0.988	0.55–1.773	**0.047**	3.978	1.016–15.584
Other	0.229	0.665	0.342–1.293	0.694	1.290	0.363–4.587
Unemployed	0.864	0.956	0.573–1.596	0.645	1.275	0.454–3.584
**Household net income**						
< €1000 (Ref.)	0.619			0.349		
€1000—< €2000	0.593	0.870	0.521–1.451	0.805	1.139	0.405–3.205
€2000—< €3000	0.264	0.736	0.429–1.261	0.267	1.892	0.613–5.835
€3000—< €4000	0.921	0.970	0.535–1.761	0.470	1.541	0.476–4.983
≥ €4000	0.699	0.878	0.454–1.697	0.115	2.907	0.770–10.975
**Start of treatment with current paediatrician**						
During the first year of life (Ref.) vs. Later	0.617	0.920	0.663–1.276	0.597	1.200	0.610–2.360
**Trust in current paediatrician**						
Very high (Ref.)	**0.003**			0.123		
Rather high	**0.006**	0.671	0.506–0.891	0.075	1.654	0.950–2.880
Low/none	**0.004**	0.522	0.334–0.814	0.590	0.787	0.330–1.878
**Constant**	0.452	1.433		0.500	1.944	

^a^degree of parent with the highest degree

^b^degrees from universities, higher education establishments and technical colleges

*Ref*. reference, *p* significance level, *OR* odds ratio, *95%-CI* confidence interval, *HL-Test* Hosmer–Lemeshow test

### Regression II

The second binomial logistic regression model is also statistically significant, χ^2^(37) = 68.004, *p* < 0.001, has a good model fit, χ^2^(8) = 14.555, *p* > 0.05 and the VIF values for all independent variables are below the threshold of 10 (ranging from 1.03 to 2.93), indicating no observable multicollinearity.

The regression shows that age (*p* = 0.011) and federal state (*p* = 0.008) have a significant influence on the actual uptake of a screening examination or check-up among those participating in AOK-Junior (see Table 4). The highest age group 12–17 years (OR: 3.270; 95%.CI: 1.507–7.094) tends to more often predict actual uptake. Moreover, living in the state of Berlin seems to be a predictor of actual uptake compared to Brandenburg (OR: 0.175; 95%-CI: 0.054–0.567) and MWP (OR: 0.175; 95%-CI: 0.058–0.533). With respect to father’s occupational status, being employed as an employee (OR: 2.014; 95%-CI: 1.033–3.924) or self-employed (OR: 3.978; 95%-CI: 1.016–15.584) is significantly associated with actual uptake (see Table 4).

## Discussion

This cross-sectional study identifies factors associated with participation in an early detection and prevention programme for children and adolescents, as well as factors associated with actual uptake of an examination, at the socio-demographic level including migration parameters, the socio-economic level and in relation to the paediatrician consulted.

### Socio-demographic parameters

The descriptive analyses show that participants are older and more likely to have a chronic disease. In addition, differences are apparent with respect to federal state, living environment and number of siblings. Regression analysis I demonstrates, however, that in terms of demographic parameters, only higher age groups are a predictor of participation in the programme. Although this result was not to be expected, as participation rates in the field of prevention generally decline with increasing age, it may be explained by the fact that within AOK-Junior more examinations are offered to children and adolescents at an older age [21]. Thus, the incentives as well as the probability to participate in the programme are likely to be greater.

With respect to regression II, age and federal state show a significant influence. Here, too, the broader selection of examinations for older children and adolescents may explain, at least partially, the larger uptake numbers. On the other hand, various studies conducted in Germany showed that participation in early detection examinations declines with increasing age and is particularly low in adolescence [33]. In the past, the participation rate for the youth examination J1 at the age of 12–14 years, which is offered as part of regular health care in Germany, varied between 32–43% depending on the study, while the participation rates for the U1-U9 at the age of 0–6 years were 96–99% [31], STMGP [6, 27, 34]. If one considers the additional preventive examination J2 for adolescents at the age of 16–17 years, the participation rate even decreased to less than 20% [33]. In this study the participation rate in J2 is 46% among AOK-Junior participants, but here too the participation rates drop significantly with increasing age from initial rates over 90% at U1-U9.

Concerning differences between federal states, it is assumed that the different design and the diversity of the examinations in the federal states affects the actual uptake. For example, the type (direct insurance billing vs. reimbursement principle) and responsibility (external service provider vs. Association of Statutory Health Insurance Physicians) of the settlement vary between the federal states. Furthermore, in the course of the project it became apparent that Berlin has the highest number of different examinations and check-ups (see Table 1), which would be in line with the results of the analysis that living in Berlin is a positive predictor for the actual uptake. In addition, there are more paediatricians in Berlin than in Brandenburg and MWP, and they are also more accessible due to the urban infrastructure, which could also have an influence.

### Migration parameters

According to the descriptive analysis, there are significant differences between participants and non-participants in terms of country of birth, migration background, and language spoken at home, while regression analysis I identifies only the language spoken at home as a predictor of participation in AOK-Junior. An earlier study confirms that children and adolescents growing up in non-German-speaking families showed lower participation rates in health check-ups compared to children and adolescents of German-speaking parents [14]. A systematic review reported a consistently lower frequency and probability of participation in preventive health programmes for children with migrant background [13], here, however, the language spoken at home was not considered separately, as is the case in most studies evaluating participation determinants. With regard to our second regression for actual uptake, no significant differences were found. This could be an indication that once the decision to participate in the programme has been made, the language spoken at home no longer matters or that the language barrier has been overcome.

### Socio-economic parameters

Descriptive results show only a significant difference in terms of occupational status of the mother and household net income. The first regression demonstrates that it is a positive predictor of participation in the AOK Junior programme if the mother is gainfully employed, while the second regression provides that it is a positive predictor for actual uptake if the father is employed as employee or self-employed. No further predictors were found in relation to socio-economic status. These results are unexpected; as several studies in the field of early detection and prevention have demonstrated that, inter alia, education and income, as well as the composite index of socio-economic status, have an influence on participation. For example, the baseline survey and the two follow-up surveys of the Study on the Health of Children and Adolescents in Germany (KiGGS) showed that children with a lower socio-economic status are significantly less likely to use preventive check-ups compared to children with a medium or high socio-economic status [12, 25, 30]. Most regional studies examining the relationship between uptake of preventive or screening examinations and education of the parents have also achieved the similar result [7, 14, 28]. The difference in results may be due to (1) different survey instruments, (2) inclusion of each dimension (educational status, occupational status, income) versus the composite index of socioeconomic status, (3) heterogeneity in sample size and composition, or (4) access to examinations (regular care vs. supplemental program). In addition, the data collection periods of most of the studies mentioned are several years old and there are no recent studies on the association of socio-economic determinants and participation in early detection examinations or prevention measures in children and adolescents.

### Treating paediatrician

The proportion with continuous paediatrician treatment since early childhood and the level of trust in the treating paediatrician differed between participating and non-participating families, according to descriptive analyses. Regression I indicates that trust in the treating paediatrician is a strong predictor for participation in the programme but no predictor for the actual uptake of an examination among AOK-Junior participants. It is common knowledge that in adult patients, trust is the foundation of a good physician–patient relationship and actually can have positive effects on patients’ continuity of care, adherence and health outcomes [3, 4, 29]. Moreover, trust in the physician can even lead to patients being more likely to take up preventive care services based on the physician's recommendation, for instance [24]. Also, in the field of paediatrics and adolescent medicine, trust between the paediatrician and the patient or the parents is of high importance [22, 23]. Reviews indicate that, especially with vaccinations, trust in the medical professionals has an influence on the decision to vaccinate [1, 31]. However, on the one hand, evidence is lacking, inter alia, on the extent to which the strength of trust in the paediatrician influences the uptake of other preventive services and early detection examinations among children and adolescents [8, 23]. On the other hand, physicians or paediatricians partly fail to use their potential influence on children and adolescents or their parents regarding health promotion or prevention by not informing families or making recommendations in this regard [26, 15]. The importance of the doctor-patient-relationship on health behaviour, especially in the field of paediatrics and adolescent medicine, has already been taken up and is or will be included in best practice examples, frameworks or guidelines to improve the physician's competences regarding e.g. communication and health promotion [5, 10, 36, 26]. This could also lead to families becoming better informed about early detection and prevention over time by their treating paediatrician and thus potentially participating more in these services.

### Limitations

This study had several limitations. First, there is a probable design-related selection bias in our study, since the questionnaire was answered on a voluntary basis, although, an attempt was made in advance to ensure that all groups were addressed equally and thus to reduce the risk of systematic non-participation and minimise possible selection bias du to non-responding. This included a stratified sample (participation, age, gender, federal state), the option of completing the questionnaire in four different languages (German, Arabic, Turkish, Russian), simple and clear wording of the invitation letter and the questionnaire itself as well as incentives and reminder. It is difficult to determine the extent of selection bias du to non-response in our study and its influence on the outcome, as a comprehensive non-responder analysis cannot be conducted due to a lack of data. Thus, the study may not be representative of all AOK-insured children and adolescents. However, potential selection bias due to non-response, e.g., socioeconomic parameters, is assumed to be the same for AOK Junior participants and non-participants, as they received the same questionnaire at the same time, so the tendency of bias is expected to be the same and the differences found between the two groups should be related to participation in the programme.

Second, causal relationships over time could not be examined due to the cross-sectional nature of our study. Third, the regression models show that the variables included contribute only partially to variance explanation, so it can be assumed that other factors play a role in the participation in the AOK-Junior programme as well as in the actual uptake of an examination. Despite these limitations, the significance of this study lies in providing actual data on a large number of potential determinants of participation in a screening and prevention programme. These results could also be transferable to other programmes in the field of paediatric and adolescent medicine and used to develop strategies to better involve previously unreached groups.

## Conclusions

The results show that children and adolescents participating in AOK-Junior differ from non-participants with regard to various factors. It turns out that although social background affects participation in the AOK Junior programme, other factors also play an important role. While age is an expected influencing factor, as there are more offers of screening examinations and check-ups in AOK-Junior with increasing age, it was unexpected that education as well as income of the parents showed no significant influence. Furthermore, not speaking German in the family is associated with a lower probability of participation, while a migration background per se is not. That trust in the treating paediatrician has such a high influence highlights how important the physician–patient relationship is also in the context of the decision in participating in an early detection and prevention programme. Therefore, there are three possible approaches to better reaching previously less reached groups and increasing the participation rate among them. First, additional offerings such as age-specific check-ups for younger children could be implemented to incentivize participation at a young age. Second, the language barrier could be overcome, for example, by offering written information material, e.g. flyers, and information on the website about the AOK-Junior programme in different languages. Here, of course, it would first be necessary to ascertain which languages would be relevant and whether other aspects relating to accessibility would also be of relevance in this context. Thirdly, it may also be possible to increase involvement of the paediatricians, on the one hand to present the programme and the potential benefits to them, and on the other hand to emphasize the importance of a solid basis of trust between them and children or adolescents as well as their parents. In addition, the role of paediatricians as disseminators of overall health promotion and prevention could also be strengthened.

With respect to the actual uptake of an examination among AOK-Junior participants, there are only differences concerning age and federal state. The former also goes along with the increasing number of examinations offered, with increasing age. The differences in the federal states can possibly be explained by the different range of examinations provided as well as by different structures of the programme design. Thus, one possibility for improvement would be to standardize the structures and services in the three federal states in order to provide children and adolescents with the same services regardless of where they live.

In terms of further research approaches, it would be of interest to further investigate the importance of trust in the paediatrician in the decision for or against a medical service, using quantitative and possibly also qualitative methods and to contribute to establishing an evidence base here. In addition, there is a general need for further research on the factors that influence participation in statutory as well as additional screening and prevention services and/or programmes for children and adolescents.

## Data Availability

The datasets analysed were used under licence for the current study. According to data protection and confidentiality clauses in the licence agreement the data is not publicly available.
